# Genomic characterization of novel IncFII-type multidrug resistant plasmids p0716-KPC and p12181-KPC from *Klebsiella pneumoniae*

**DOI:** 10.1038/s41598-017-06283-z

**Published:** 2017-07-19

**Authors:** Jiao Feng, Zhe Yin, Qiangyuan Zhao, Yachao Zhao, Defu Zhang, Xiaoyuan Jiang, Weili Wu, Weijun Chen, Hui Wang, Yajun Song, Yigang Tong, Jinglin Wang, Yanjun Li, Dongsheng Zhou

**Affiliations:** 1 State Key Laboratory of Pathogen and Biosecurity, Beijing Institute of Microbiology and Epidemiology, Beijing, 100071 China; 2 Laboratory Department, Navy General Hospital, Beijing, 100048 China; 3 Beijing Institute of Genomics, Chinese Academy of Sciences, Beijing, 100029 China; 4 College of Food Science and Project Engineering, Bohai University, Jinzhou, 121013 China

## Abstract

This study aimed to genetically characterize two fully-sequenced novel IncFII-type multidrug resistant (MDR) plasmids, p0716-KPC and p12181-KPC, recovered from two different clinical *Klebsiella pneumoniae* isolates. p0716-KPC and p12181-KPC had a very similar genomic content. The backbones of p0716-KPC/p12181-KPC contained two different replicons (belonging to a novel IncFII subtype and the Rep_3 family), the IncFII_K_ and IncFII_Y_ maintenance regions, and conjugal transfer gene sets from IncFII_K_-type plasmids and unknown origins. p0716-KPC and p12181-KPC carried similar three accessory resistance regions, namely ΔTn*6209*, a MDR region, and the *bla*_KPC-2_ region. Resistance genes *bla*_KPC-2_, *mph(A)*, *strAB*, *aacC2*, *qacEΔ1*, *sul1*, *sul2*, and *dfrA25*, which are associated with transposons, integrons, and insertion sequence-based mobile units, were located in these accessory regions. p0716-KPC carried two additional resistance genes: *aphA1a* and *bla*_TEM-1_. Together, our analyses showed that p0716-KPC and p12181-KPC belong to a novel IncFII subtype and display a complex chimeric nature, and that the carbapenem resistance gene *bla*_KPC-2_ coexists with a lot of additional resistance genes on these two plasmids.

## Introduction

Carbapenemases can be divided into three main categories: Ambler class A serine β-lactamases, class B metallo-β-lactamases, and the class D OXA group. *Klebsiella pneumoniae* carbapenemase (KPC) is a class A β-lactamase that was initially discovered in the USA in 1996. It has since disseminated worldwide among Enterobacteriaceae, *Pseudomonas*, and *Acinetobacter* species, with *K. pneumoniae* being the most common species harboring *bla*_KPC_ genes^1, 2^. KPC-producing bacteria are becoming endemic in certain hospitals, and are responsible for increasing numbers of outbreaks in healthcare facilities. KPC confers resistance or decreased susceptibility to almost all β-lactams, and KPC-producing isolates are often resistant to many other non-β-lactam drugs because of the co-occurrence of *bla*_KPC_ with other classes of resistance gene. This multidrug resistance (MDR) leaves few available options for antimicrobial treatment, and thereby results in high mortality rates^3^.

The *bla*_KPC_ genes have been found on IncFII-related plasmids such as pKPHS2 (GenBank accession number CP003224)^4^ and pKPC-LK30 (accession number KC405622)^5^ from *K. pneumoniae*. Conjugative IncFII_K_ plasmid pKPHS2 has the core IncFII_K_ backbone regions for plasmid replication (*repA*_IncFIIK5_), maintenance (*parAB*, *stbAB*, *umuCD*, *psiAB*, *ardAB*, and *relBE*), and conjugal transfer (*tra* and *trb*), as well as additional replication genes *repA2*_IncFIB-like_ and *repB*_Rep_3-family/pKPHS2_. pKPC-LK30 lacks plasmid conjugal transfer regions, which might result in it being nonconjugative. The pKPC-LK30 backbone is composed of a single replication gene, *repB*_Rep_3-family/pKPHS2_, a 36-kb IncFII_Y_-type maintenance region homologous to a portion of the pKPHS2 maintenance regions, and a 16-kb IncFII_Y_-type plasmid maintenance region found in pKOX_NDM1, which is a *bla*_NDM-1_-carrying IncFII_Y_-type plasmid from *Klebsiella oxytoca* isolated in China^6^. Although highly unusual, these backbone components can function together to promote the replication and stability of pKPC-LK30 in *K. pneumoniae*.

This work presents the complete sequences of two novel MDR plasmids, p0716-KPC and p12181-KPC, from *K. pneumoniae* strains isolated from China (Table 1). The two closely related plasmids belong to a novel IncFII subtype, and displayed a complex chimeric nature with respect to both the plasmid backbone (closely related to pKPHS2 and pKPC-LK30) and the accessory resistance regions. Co-occurrence of *bla*_KPC-2_ (carbapenem resistance) with *mph(A)* (macrolide resistance), *strAB* and *aacC2* (aminoglycoside resistance), *qacEΔ1* (quaternary ammonium compound resistance), *sul1* and *sul2* (sulphonamide resistance), and *dfrA25* (trimethoprim resistance) was observed in both plasmids.

**Table 1:** Major features of p0716-KPC and p12181-KPC and their antibiotic resistance genes and host bacteria.

Category	Plasmid					
	p0716-KPC			p12181-KPC		
Accessory resistance regions	∆Tn*6029*	MDR region	*bla*_KPC-2_ region	∆Tn*6029*	MDR region	*bla*_KPC-2_ region
Resistance genes	*strAB*, and *sul2*	*aphA1a*, *ΔtmrB*, *aacC2*, *mph(A)*, *sul1*, *qacEΔ1*, and *dfrA25*	*bla*_TEM-1_, and *bla*_KPC-2_	*strAB*, and *sul2*	*mph(A)*, *sul1*, *qacEΔ1*, and *dfrA25*	*ΔtmrB*, *aacC2*, and *bla*_KPC-2_
Host bacterium	*K. pneumoniae* 0716			*K. pneumoniae* 12181		
Bacterial isolation	Recovered from ascitic fluid from Patient 1 in Hospital 1			Recovered from sputum from Patient 2 in Hospital 2		

## Results

### Clinical cases

Patient 1 was a 59-year-old male admitted to Hospital 1 in October 2013, where he was diagnosed with a cardiac carcinoma. Nosocomial intra-abdominal infection occurred, and *K. pneumoniae* 0716 was isolated from ascitic fluid. The patient received intravenous administration of tigecycline, and the patient’s acute condition significantly improved.

Patient 2 was an 87-year-old man with chronic obstructive pulmonary disease, chronic bronchitis, pulmonary emphysema, coronary heart disease, and pancreatic carcinoma. Acute pancreatitis and peritonitis developed during treatment in Hospital 2 in September 2013. *K. pneumoniae* 12181 was isolated from a sputum sample, and the patient was treated with intravenous administration of meropenem plus ciprofloxacin. However, treatment was unsuccessful and the patient died.

Strains 0716 and 12181 were resistant to multiple antibiotics, including ampicillin, β-lactamase inhibitors (amoxicillin/clavulanic acid and piperacillin/tazobactam), cephalosporins (cefazolin and ceftriaxone), carbapenems (imipenem and meropenem), aztreonam, macrodantin, fluoroquinolones (ciprofloxacin and levofloxacin), aminoglycosides (amikacin and tobramycin), and trimethoprim/sulfamethoxazole, but remained susceptible to tetracycline (data not shown).

### Overview of p0716-KPC and p12181-KPC

PCR screening and sequencing indicated the presence of *bla*_KPC-2_, but none of the other carbapenemase genes tested for, in strains 0716 and 12181. The *bla*_KPC_ markers could be transferred from strains 0716 and 12181 into TOP10 through electroporation, generating the *E. coli* electroporants 0716-KPC-TOP10 and 12181-KPC-TOP10, respectively. Subsequent high-throughput sequencing indicated that these two electroporants contained the *bla*_KPC-2_-carrying plasmids p0716-KPC and p12181-KPC, respectively. p0716-KPC but not p12181-KPC could be transferred from the respective strains 0716 and 12181 into EC600 through conjugation. In the negative control experiments using only the donor or the recipient, no colonies were observed on the agar plates containing indicated antibiotics, excluding the possibility of antibiotic resistance due to spontaneous mutation.

As expected, the resulting *E. coli* transformants and transconjugant demonstrated class A carbapenemase activity, and were resistant to ampicillin, amoxicillin/clavulanic acid, piperacillin/tazobactam, cefazolin, ceftriaxone, imipenem, meropenem, and aztreonam, but remained susceptible to macrodantin (data not shown).

**Figure 1:**
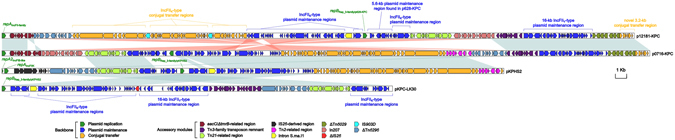
Linear comparison of sequenced plasmids. Genes are denoted by arrows and are colored based on gene function classification. Shaded regions denote regions of homology (>95% nucleotide similarity). The sequences of p0716-KPC and p12181-KPC were determined in this study, while those of pKPC-LK30 and pKPHS2 are derived from GenBank.

Genome sequencing confirmed that p0716-KPC and p12181-KPC were circular DNA molecules, 143,538 bp and 140,089 bp in length, with 192 and 188 predicted open reading frames, respectively (Figure S1). The modular structure of each plasmid was composed of the backbone region, as well as multiple separate accessory modules inserted within the backbone (Figure S1). p0716-KPC and p12181-KPC shared 97% query coverage with a maximum nucleotide identity of 99% (Fig. 1).

### Backbone regions of p0716-KPC and p12181-KPC

Two representative *bla*_KPC-2_-carrying plasmids pKPHS2 and pKPC-LK30 were included in the genomic comparison with p0716-KPC and p12181-KPC (Fig. 1). p0716-KPC and p12181-KPC were most closely related to pKPHS2 (67% coverage and 99% nucleotide identity; last accessed on August 15, 2015). pKPC-LK30 was selected because it contains a large plasmid maintenance region that is not found in pKPHS2 but was identified in p0716-KPC and p12181-KPC (see below).

The backbones of p0716-KPC and p12181-KPC were almost identical with respect to genetic content. p0716-KPC and p12181-KPC both contained two novel replicons, including a *repB*_Rep_3-family/p0716-KPC_ gene and a *repA*_IncFII-family_ gene, both of which were very different from their counterparts in pKPHS2 and pKPC-LK30. The *repA*_IncFII-family_ gene was most closely related to the IncFII plasmid pEA49-KPC (accession number KU318419) with a nucleotide identity of 94% (last accessed on December 25, 2016). p0716-KPC and p12181-KPC also contained the complete 16-kb IncFII_Y_-type pKOX_NDM1 maintenance region, and almost the entire non-redundant IncFII_K_-type maintenance region found in both pKPHS2 and pKPC-LK30 (Fig. 1). Compared with pKPHS2 and pKPC-LK30, p0716-KPC and p12181-KPC contained two unique backbone regions: a 5.6-kb plasmid maintenance region connected to the *repB*_Rep_3-family/p0716-KPC_ gene, and a novel 3.2-kb conjugal transfer region contained the *pld* gene (conjugal transfer endonuclease). This 3.2-kb region was most closely related (93% query coverage and 98% maximum nucleotide identity; last accessed on December 25, 2016) to the MOB_F_ family plasmid pEA49-KPC (Fig. 1).

p0716-KPC and p12181-KPC belonged to a novel IncFII subtype since it contained a novel *repA* gene belonging to the IncFII family, together with IncFII_K_/II_Y_-type maintenance regions. Based on the three key regulatory DNA transfer genes *traM*, *traJ*, and *finO* and the ATPase gene *traC*, all of which encoded key proteins of a type IV secretion system, p0716-KPC and p12181-KPC were assigned into the subgroup A of the IncF/MOB_F12_ group^7^.

### Accessory regions of p0716-KPC and p12181-KPC

**Figure 2:**
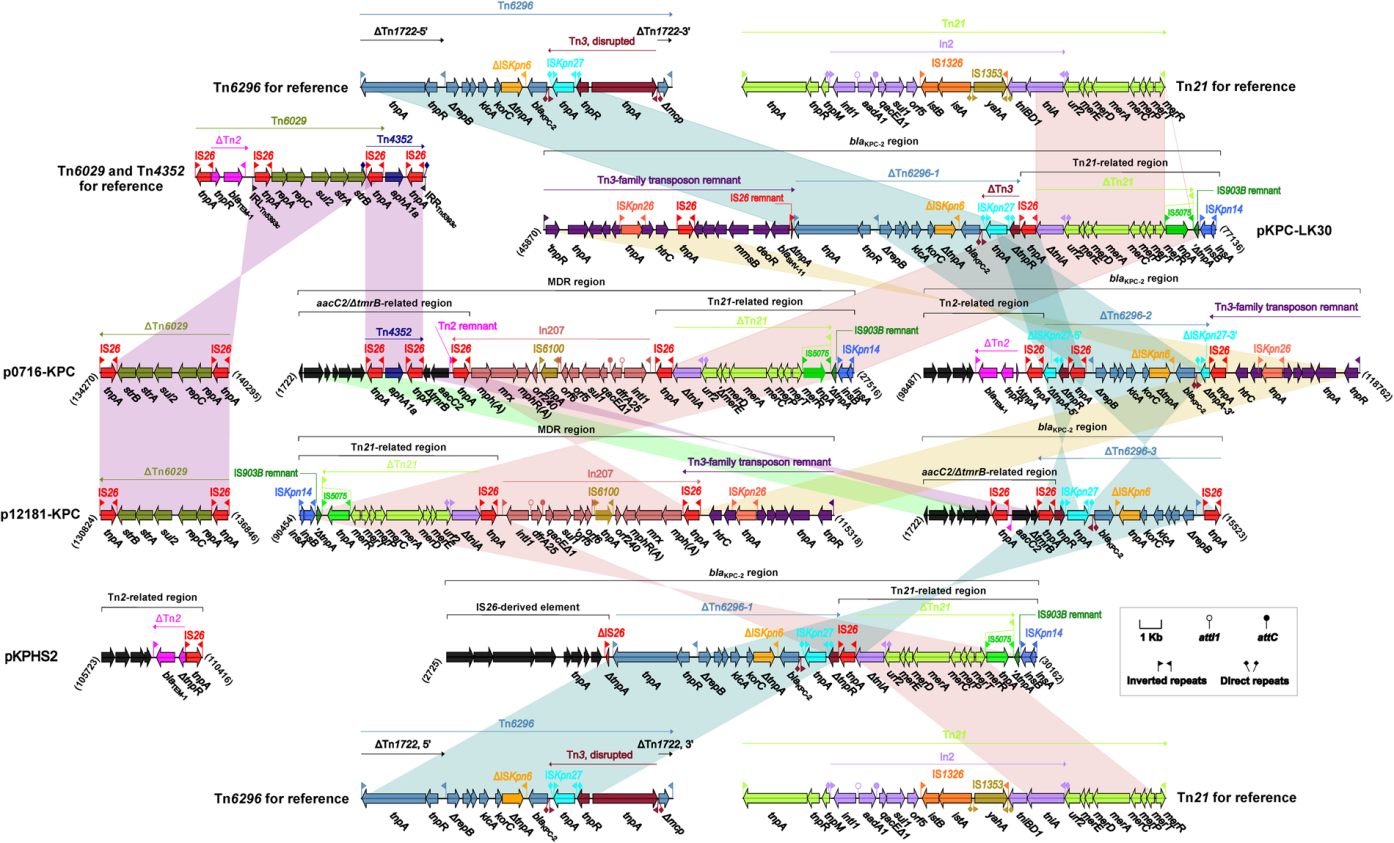
Organization and alignment of resistance regions. The genetic organization of the resistance regions from p0716-KPC, p12181-KPC, pKPC-LK30, and pKPHS2 is shown, and relevant mobile elements are included for reference. Genes are denoted by arrows and are colored based on gene function classification. Shaded regions denote regions of homology (>95% nucleotide similarity).

p0716-KPC contained three accessory modules in total, namely ΔTn*6029*, the MDR region, and the *bla*_KPC-2_ region, which were inserted at different sites in the backbone (Fig. 2). ΔTn*6029* is a partial fragment of the IS*26*-based composite transposon Tn*6029* ^8^, and comprises a *strAB* module flanked by two inverted IS*26* elements. As initially characterized in the IncHI1 plasmid pSRC27-H, Tn*6029* and Tn*4352* are two overlapping transposons likely generated from complex recombination events between IS*26*, Tn*2*, and Tn*5393c* ^8^.

The MDR region was further divided into an *aacC2*/*ΔtmrB*-related region, In207, and a Tn*21*-related region. The *aacC2*/*ΔtmrB*-related region harbored the *aphA1a* (aminoglycoside resistance)-carrying Tn*4352* ^8^, which lacked target site duplication signals of transposition. Tn*4352* was further connected to a region composed of *ΔtmrB*, *aacC2*, and a 135-bp Tn*2* remnant containing its inverted repeat right (IRR), resulting in truncation of *tmrB* (tunicamycin resistance). The association of *aacC2-tmrB* with Tn*2* and IS*26*, as observed in pCTX-M3 and pU302L, constitutes one example of the multifarious *aacC2*-harboring structures^9^. In207 was identified in p0716-KPC and p12181-KPC, and is an In4-like integron^10^ containing a single *dfrA25* cassette. Notably, In207 is connected to the macrolide resistance unit IS*26*-*mph(A)*-*mrx*-*mphR(A)*-IS*6100*, a structure frequently associated with class 1 integrons^9^, at its 3ʹ region. This likely occurred through IS*6100*-mediated recombination, and resulted in deletion of the inverted repeat terminal (IRt). In207 from p0716-KPC and p12181-KPC appeared to be more complete than the prototype In207 (GenBank accession number AB280920), which is only an integron cassette array fragment. The Tn*21*-related region was organized sequentially as follows: IS*26*, ∆Tn*21*, a 288-bp IS*903B* remnant, and IS*Kpn14*. Tn*21*, a Tn*3*-family unit transposon, contained the core transposition module *tnpA* (transposase)-*tnpR* (resolvase), *tnpM*, inserted integron In2, *urf2*, and the mercuric resistance (*mer*) operon. The transposon was flanked by 38-bp IRL (inverted repeat left) and IRR sequences, and the In2 insertion was shown to disrupt a presumed ancestral *urf2M* gene, resulting in *urf2* and *tnpM* ^11^. The ∆Tn*21* element from p0716-KPC was composed of *ΔtniA*_In2_, the *mer* operon, and the IS*5075*-disrupted IRR_Tn*21*_. The IS*1111*-family element IS*5075* targets the terminal inverted repeats of the Tn*21*-subgroup transposons of the Tn*3* family^12^.

The *bla*_KPC-2_ region consisted of a Tn*2*-related region, ΔTn*6296-2*, and a Tn*3*-family transposon remnant. Tn*6296* (designated in this work) was originally identified in the MDR plasmid pKP048^13^ from *K. pneumoniae*, and is generated from the insertion of a core *bla*_KPC-2_ genetic platform (∆Tn*3*:IS*Kpn27-bla*_KPC-2_ *-*ΔIS*Kpn6-korC-orf6*-*klcA-*Δ*repB*) into Tn*1722*, resulting in truncation of *mcp* ^14^. Various Tn*6296* derivatives with deletions, insertions, and rearrangements at different sites, such as ΔTn*6296-1* in pKPHS2/pKPC-LK30, ΔTn*6296-2* in p0716-KPC, and ΔTn*6296-3* in p12181-KPC, have been identified in KPC-encoding plasmids from *K. pneumoniae* strains isolated in China^14^. A four-gene cluster coding for proteins of unknown function was located at the 5ʹ end of the Tn*2*-related region, followed by a partial *bla*_TEM-1_ (β-lactam resistance)-carrying Tn*2* ^15^ region, plus IS*26*. The cryptic Tn*3*-family transposon remnant contained a typical 38-bp IRL element, a core transposition module (*tnpA*-*tnpR*), and inserted IS elements IS*Kpn26* and IS*26*.

p12181-KPC also contained ΔTn*6029*, the MDR region, and the *bla*_KPC-2_ region, which resembled their counterparts in p0716-KPC. Tn*4352* in the MDR region and the Tn*2*-related region in the *bla*_KPC-2_ locus were not found in p12181-KPC. Therefore, p0716-KPC contained two additional resistance genes, *aphA1a* and *bla*_TEM-1_, compared with p12181-KPC. Notably, *aphA1a* and *bla*_TEM-1_ are redundant determinants accounting for resistance to aminoglycosides and β-lactams, respectively, in p0716-KPC. In addition, extensive rearrangement of large fragments was observed not only within, but between the MDR region and the *bla*_KPC-2_ region of p12181-KPC relative to p0716-KPC. These rearrangements were likely promoted by IS*26*-based replicative transposition^16^ as multiple copies of IS*26* were identified in these two accessory regions. p12181-KPC still maintained three small accessory regions: two IS*903D* copies and group IIB retro-transposable intron S.ma.I1, none of which were found in p0716-KPC. One copy of IS*903D* was inserted into a region between *trbF* and *trbB*, while *traV* (an essential gene encoding a core protein of type IV secretion system) was disrupted by a second copy, rendering p12181-KPC non-conjugative.

pKPC-LK30 contained a single accessory resistance region (the *bla*_KPC-2_ region) containing two antibiotic resistance genes: *bla*_KPC-2_ and *bla*_SHV-11_ (Fig. 2). pKPHS2 carried two accessory resistance regions, namely the *bla*_KPC-2_ region and the Tn*2*-related region, each of which contained a single antibiotic resistance gene (*bla*_KPC-2_ and *bla*_TEM-1_, respectively) (Fig. 2). All these accessory resistance regions were genetically related to their counterparts from p0716-KPC and p12181-KPC. Compared with pKPC-LK30 and pKPHS2, p0716-KPC and p12181-KPC appear to have acquired many more accessory regions containing several additional resistance genes.

## Discussion

The *bla*_KPC_ genes are largely associated with Tn*4401* and Tn*6296* ^14^, which constitute the core *bla*_KPC_ genetic environments. *bla*_KPC_-carrying Tn*4401b* and its close derivatives are frequently found on plasmids from bacteria isolated in European and American countries^17–19^. Tn*4401* is rarely found in China^20^, with Tn*6296* and its derivatives more frequently identified as the *bla*_KPC_ platforms, such as those located in pKP048^13^, pKPHS2^4^, pKPC-LK30^5^, and p0716-KPC and p12181-KPC (this study).

*bla*_KPC_ genes are also commonly identified in plasmids belonging to various incompatibility groups, including IncF, IncI, IncA/C, IncN, IncX, IncR, IncP, IncU, IncW, IncL/M, and ColE, ranging in size from 10–300 kb^21^. The IncF replicons can be classified into the groups FIA, FIB, FIC, and FII^22^. The IncFII plasmids are commonly low copy number plasmids and carry the primary FII replicon, often in association with additional replicons such as FIA and FIB. Moreover, the FII replicons can be further divided into various subtypes, including II_K_, FII_Y_, and FII_S_, generating many compatible variants that can be used to overcome the incompatibility barrier with incoming plasmids^22^.

The IncFII plasmid family can replicate in many different enterobacterial species, and is clearly playing an important role in the dissemination of antimicrobial resistance genes, including *bla*_KPC_, amongst Enterobacteriaceae^21^. The *bla*_KPC-3_-carrying IncFII_K_ plasmid pKpQIL and its close derivatives have spread in European and American countries^23^. *bla*_KPC-2_-carrying IncFII_K_ plasmids from China, such as pKP048 and pKPHS2, have very similar core backbone regions but limited overall sequence similarity to pKpQIL. It seems that the pKpQIL-like and pKP048-like plasmids followed distinct evolutionary routes after separating from their common ancestor.

Recovery of the closely related plasmids p0716-KPC and p12181-KPC from two independent cases of nosocomial infection from two different hospitals indicates the potential of trans-regional spread and circulation of these plasmids in hospital settings. p0716-KPC and p12181-KPC belong to a novel IncFII subtype, and display a complex chimeric nature, as observed within the backbone as well as the accessory resistance regions. The replication and stable inheritance of these two plasmids is likely promoted by the coordinated action of the IncFII and Rep_3-family replicons and the IncFII_K_ and IncFII_Y_ maintenance gene sets, respectively.

Production of KPC-2 makes strains containing p0716-KPC or p12181-KPC resistant to almost all β-lactams, including carbapenems. The situation is exacerbated by the presence of five additional classes of antibiotic resistance genes [*mph*(A), *strAB* and *aacC2*, *qacEΔ1*, *sul1* and *sul2*, and *dfrA25*] on these two plasmids. The accumulation of various antibiotic resistance genes on a plasmid have resulted from complex horizontal genetic transfer events under selection pressure of multiple antibiotics, and a bacterium will become resistant to multiple antibiotics at once by picking up such a MDR plasmid.

p0716-KPC is conjugative and contains the complete IncFII_K_ conjugal transfer gene content, while p12181-KPC has become non-conjugative likely because of the presence of multiple genetic lesions in the conjugal transfer regions. Non-transmissible plasmids rely largely on vertical transmission to be maintained in populations. Although classical models of plasmid evolution predict that conjugation is necessary for plasmid maintenance, it has been found that compensatory adaptation to ameliorate the cost of plasmid carriage coupled to rare (positive) selection for plasmid-encoded antibiotic resistance is sufficient to stabilize non-transmissible plasmids, explaining why non-conjugative plasmids are common^24^.

## Materials and Methods

### Bacterial strains and identification

The use of human specimens and all related experimental protocols was approved by the Committee on Human Research of all the institutions (Beijing Institute of Microbiology and Epidemiology, the 307th Hospital of the People’s Liberation Army, and Navy General Hospital), and was carried out in accordance with the approved guidelines. Informed consent was obtained from patients where indicated. Our research was carried out in accordance with the Declaration of Helsinki.

Imipenem-non-susceptible *K. pneumoniae* strains 0716 and 12181 were isolated from two inpatients with hospital-acquired infections from two different public hospitals, and there was no epidemiological link between the two patients. Bacterial species identification was performed by 16 S rRNA gene sequencing^25^. The major plasmid-borne carbapenemase genes were screened by PCR^26^. All PCR amplicons were sequenced on an ABI 3730 Sequencer using the PCR primers.

### Plasmid conjugal transfer

Plasmid conjugal transfer experiments were carried out using rifampin-resistant *Escherichia coli* strain EC600 as the recipient, and *K. pneumoniae* strains 0716 and 12181 as donors. Aliquots (3 ml) of overnight culture of each donor and recipient strain were mixed, harvested, and resuspended in 80 μl of Brain Heart Infusion (BHI) broth (BD Biosciences). The mixtures were spotted on 1 cm^2^ hydrophilic nylon membrane filters with a 0.45-µm pore size (Millipore), which were then placed on BHI agar (BD Biosciences) plates and incubated at 37 °C for 12–18 h. Bacteria were washed from the filter membranes and spotted on Muller-Hinton (MH) agar (BD Biosciences) plates containing 1 mg/ml rifampin and 2 μg/ml imipenem for selection of *bla*_KPC_-positive *E. coli* transconjugants.

### Plasmid electroporation

To prepare competent *E. coli* TOP10 cells for plasmid electroporation, 200 ml of overnight culture in Super Optimal Broth (SOB) at an optical density (OD_600_) of 0.4–0.6 were washed three times with electroporation buffer (0.5 M mannitol and 10% glycerol), and then concentrated into a final volume of 2 ml. A 1-µg aliquot of plasmid DNA, isolated from strain 0716 or 12181 using a Qiagen Plasmid Midi Kit, was mixed with 100 μl of competent cells for electroporation at 25 μF, 200 Ω, and 2.5 kV. Immediately following electroporation, cells were suspended in 500 μl of SOB, and an appropriate aliquot was spotted on an SOB agar plate containing 2 μg/ml imipenem for selection of *bla*_KPC_-positive *E. coli* transformants.

### Detection of carbapenemase activity

Activity of class A/B/D carbapenemases in bacterial cell extracts was determined via a modified CarbaNP test^26^. Briefly, overnight bacterial culture in MH broth was diluted 1:100 into 3 ml of fresh MH broth, and then incubated at 37 °C with shaking at 200 rpm to an OD_600_ of 1.0–1.4. If required, ampicillin was used at 200 μg/ml. Bacterial cells were harvested from 2 ml of the above culture and washed twice with 20 mM Tris-HCl (pH 7.8). Cell pellets were resuspended in 500 μl of 20 mM Tris-HCl (pH 7.8), lysed by sonication, and then pelleted by centrifugation at 10000 × *g* for 5 min at 4 °C. Aliquots (50 µl) of the supernatant (the enzymatic bacterial suspension) were individually mixed with 50 μl of substrates I–V, followed by incubation at 37 °C for a maximum of 2 h. The substrates consisted of: (I) 0.054% phenol red, 0.1 mM ZnSO_4_ (pH 7.8); (II) 0.054% phenol red, 0.1 mM ZnSO_4_ (pH 7.8), 0.6 mg/μl imipenem; (III) 0.054% phenol red, 0.1 mM ZnSO_4_ (pH 7.8), 0.6 mg/μl imipenem, 0.8 mg/μl tazobactam; (IV) 0.054% phenol red, 0.1 mM ZnSO_4_ (pH 7.8), 0.6 mg/μl imipenem, 3 mM EDTA (pH 7.8); (V) 0.054% phenol red plus 0.1 mM ZnSO_4_ (pH 7.8), 0.6 mg/μl mg imipenem, 0.8 mg/μl tazobactam, 3 mM EDTA (pH 7.8).

### Antimicrobial susceptibility testing

Antimicrobial susceptibility testing was conducted using the VITEK 2 system (bioMérieux) according to the manufacturer’s instructions, and interpreted as per the Clinical and Laboratory Standards Institute guidelines^27^.

### Plasmid sequencing and annotation

Plasmid DNA was isolated from *E. coli* transformants using a Qiagen Large Construct Kit, and then sequenced from a paired-end library with an average insert size of 500 bp, and a mate-pair library with average insert size of 5,000 bp, using an Illumina MiSeq sequencer. The circled DNA contigs were assembled using Newbler 2.6^28^. Open reading frames and pseudogenes were predicted using RAST 2.0^29^ combined with BLASTP/BLASTN^30^ searches against the UniProtKB/Swiss-Prot^31^ and RefSeq^32^ databases. Annotation of resistance genes, mobile elements, and other features was carried out using CARD^33^, ResFinder^34^, ISfinder^35^, INTEGRALL^36^, and the Tn Number Registry^37^. Multiple and pairwise sequence comparisons were performed using MUSCLE 3.8.31^38^ and BLASTN, respectively. Gene organization diagrams were drawn in Inkscape 0.48.1.

### Nucleotide sequence accession numbers

The complete nucleotide sequences of p0716-KPC and p12181-KPC were submitted to GenBank under accession numbers KY270849 and KY270850, respectively.

## Electronic supplementary material

Figure S1
