# Supplementary material for: Genomic characterization of novel IncFII-type multidrug resistant plasmids p0716-KPC and p12181-KPC from Klebsiella pneumoniae
Source: Sci Rep. 2017 Jul 19;7:5830. doi: 10.1038/s41598-017-06283-z (PMC5517477; doi:10.1038/s41598-017-06283-z)
Supplement: Supplementary file 1 — Figure S1 [file 41598_2017_6283_MOESM1_ESM.pdf]

Genomic characterization of novel IncFII-type multidrug resistant plasmids p0716-KPC and p12181-KPC from *Klebsiella pneumoniae*

Jiao Feng[1], Zhe Yin[1], Qiangyuan Zhao[2], Yachao Zhao[1], Defu Zhang[1, 4], Xiaoyuan Jiang[1], Weili Wu[3], Weijun Chen[3], Hui Wang[1], Yajun Song[1], Yigang Tong[1], Jingling Wang[1], Yanjun Li[2], Dongsheng Zhou[1]

1, State Key Laboratory of Pathogen and Biosecurity, Beijing Institute of Microbiology and Epidemiology, Beijing 100071, China; 2, Laboratory Department, Navy General Hospital, Beijing 100048, China; 3, Beijing Institute of Genomics, Chinese Academy of Sciences, Beijing, 100029, China; 4, College of Food Science and Project Engineering, Bohai University, Jinzhou 121013, China

Short running title: p0716-KPC and p12181-KPC from *K. pneumoniae*

Jiao Feng and Zhe Yin contributed equally to this work.

Corresponding authors:

Dongsheng Zhou, E-mail: dongshengzhou1977@gmail.com

Yanjun Li, E-mail: liyanjun1230@163.com

Jinglin Wang, E-mail: wjlwjl0801@sina.com

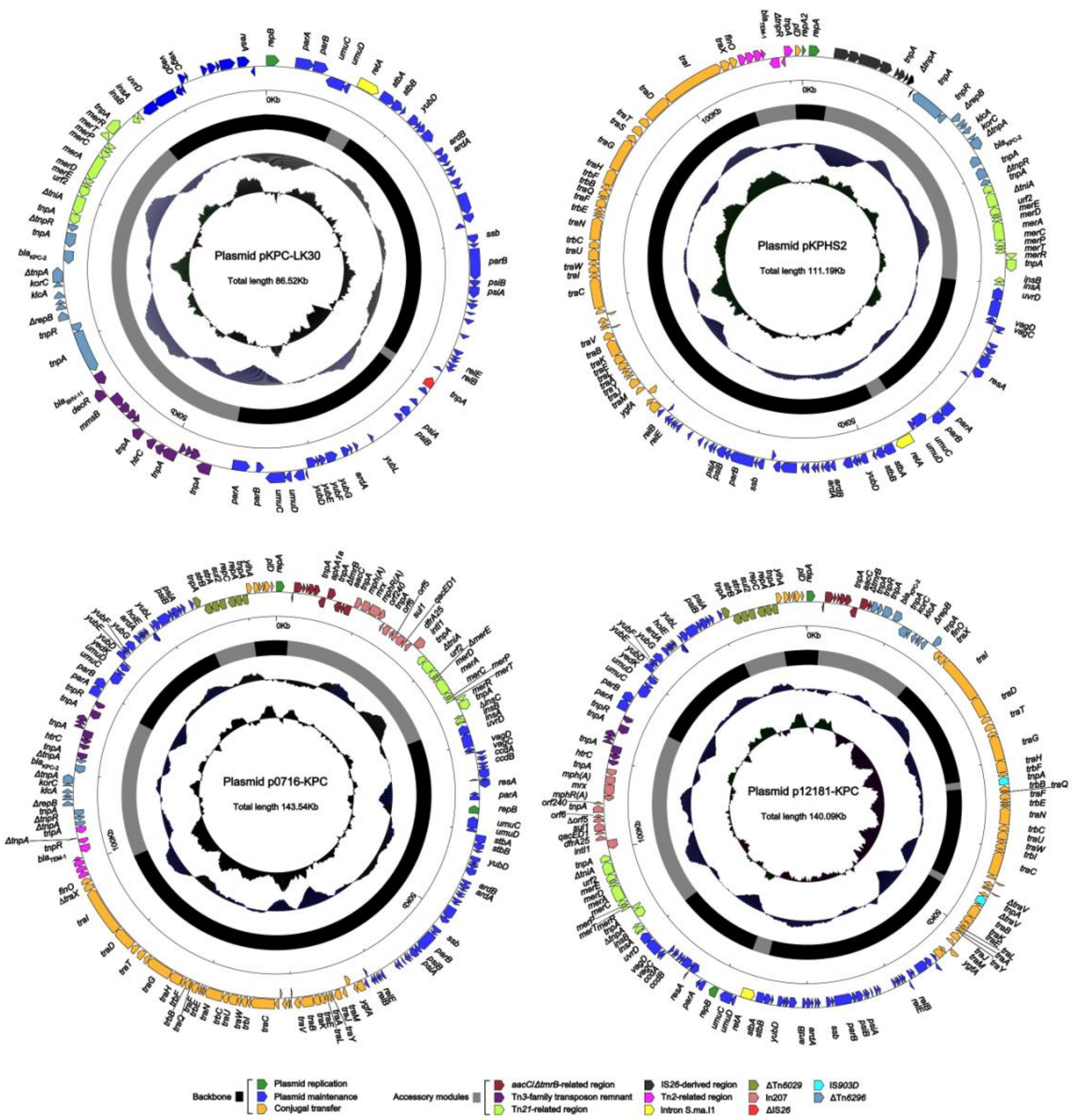

Figure S1. Schematic maps of sequenced plasmids. Genes are denoted by arrows and are colored based on gene function classification. The innermost circle presents GC-Skew [(G-C)/(G+C)] with a window size of 500 bp and a step size of 20 bp. The following circle presents GC content. Backbone and accessory module regions are also shown.
